# Supplementary material for: Impact of the Pre-Transplant Circulatory Supportive Strategy on Post-Transplant Outcome: Double Bridge May Work
Source: J Clin Med. 2021 Oct 13;10(20):4697. doi: 10.3390/jcm10204697 (PMC8539306; doi:10.3390/jcm10204697)
Supplement: Supplementary file 1 [file jcm-10-04697-s001.zip › jcm-1388759-supplementary.pdf]

Table S1. Key hepatic and renal biomarkers in the study group after transplantation.

	Without MCS	With MCS	*p*	Total
Patient, n (%)	115 (45.8)	136 (54.2)		251
Total Bilirubin, (mg/dL ± SD)				
Time of index admission	1.9 ± 1.4	2.6 ± 2.8	0.042	2.3 ± 2.3
Pre-HTx	2.6 ± 8.3	3.1 ± 4.3	0.551	2.9 ± 6.3
1-month after HTx	1.4 ± 4.9	1.4 ± 2.7	0.884	1.4 ± 3.9
3-month after HTx	0.7 ± 0.3	1.3 ± 4.4	0.187	1.0 ± 3.2
6-month after HTx	0.8 ± 0.5	0.7 ± 0.4	0.025	0.8 ± 0.4
9-month after HTx	0.8 ± 0.4	0.7 ± 0.3	0.011	0.7 ± 0.3
1-year after HTx	0.8 ± 0.5	0.7 ± 0.4	0.200	0.8 ± 0.4
3-year after Htx	0.7 ± 0.3	0.7 ± 0.3	0.617	0.7 ± 0.3
Creatinine, (mg/dL ± SD)				

Time of index admission	1.6 ± 1.7	1.7 ± 1.0	0.394	1.6 ± 1.3
Pre-HTx	1.5 ± 1.6	1.5 ± 1.0	0.941	1.5 ± 1.3
1-month after HTx	1.5 ± 1.4	1.5 ± 1.0	0.796	1.5 ± 1.2
3-month after HTx	1.5 ± 1.2	1.8 ± 1.4	0.110	1.6 ± 1.3
6-month after HTx	1.7 ± 1.8	1.9 ± 1.6	0.388	1.8 ± 1.7
9-month after HTx	1.7± 1.7	1.9 ± 1.7	0.306	1.8± 1.7
1-year after HTx	1.6 ± 1.7	1.9 ± 1.7	0.300	1.7 ± 1.7
3-year after Htx	1.7± 2.0	1.7± 1.4	0.915	1.7 ± 1.8

HTx: heart transplantation, MCS: mechanical circulatory support, SD: standard deviation.

Table S2. Creatinine and Bilirubin data of MCS bridge to HTx subgroups.

	IABP	ECMO	ECMO-VAD	VAD	*p*	Total

Patient, n (%)	15 (11)	33 (24.3)	59 (43.4)	29 (21.3)		136
**Creatinine, (mg/dL mean± SD)**						
Time of admission	1.7 ± 1.2	1.8 ± 0.9	1.6 ± 0.9	1.7 ± 1.1	*0.872*	1.7 ± 1.0
Time of MCS	1.6 ± 1.0	2.1 ± 1.0	1.7 ± 1.0 (E)<br>1.8 ± 1.0(V)	2.1 ± 1.2	*0.487*	1.9 ± 1.1
Pre-HTx	1.3 ± 0.8	1.8 ± 1.2	1.4 ± 1.1	1.3 ± 0.9	*0.251*	1.5 ± 1.0
1-month after HTx	1.6 ± 1.1	1.4 ± 0.7	1.5 ± 1.0	1.4 ± 1.2	*0.927*	1.5 ± 1.0
3-month after HTx	1.9 ±1.4	1.7 ± 0.8	1.9 ± 1.7	1.6 ± 1.4	*0.699*	1.8 ± 1.4
6-month after HTx	2.0 ± 2.2	1.9 ± 1.9	2.0 ± 1.6	1.8 ± 1.3	*0.955*	1.9 ± 1.6
9-month after HTx	2.2 ± 3.2	1.6 ± 0.7	1.8 ± 1.4	2.1 ± 2.1	*0.771*	1.9 ± 1.7
1-year after HTx	2.1 ± 2.7	1.5 ± 0.6	1.8 ± 1.5	2.0 ± 2.0	*0.769*	1.9 ± 1.7
3-year after HTx	1.3 ± 0.6	1.5 ± 0.6	1.6 ± 1.1	2.2 ± 2.5	*0.496*	1.7 ± 1.4
**Bilirubin, (mg/dL mean ± SD)**						

Time of admission	2.6 ± 1.8	3.1 ± 2.8	2.7 ± 3.5	1.9 ± 1.5	*0.551*	2.6 ± 2.8
Time of MCS	3.4 ± 2.3	3.7 ± 4.7	2.3 ± 1.5	2.5 ± 1.8	*0.298*	2.9 ± 3.0
Pre-HTx	1.5 ± 1.1	5.8 ± 6.9	2.5 ± 2.8	2.2 ± 2.2	*0.000*	3.1 ± 4.3
1-month after HTx	0.6 ± 0.3	2.6 ± 4.9	1.0 ± 1.5	1.2 ± 1.8	*0.056*	1.4 ± 2.7
3-month after HTx	0.6 ±0.2	2.6 ± 8.8	1.0 ±2.3	0.7 ± 0.5	*0.378*	1.3 ± 4.4
6-month after HTx	0.7 ± 0.3	0.9 ±0.5	0.7 ± 0.3	0.7 ± 0.3	*0.179*	0.8 ± 0.4
9-month after HTx	0.9 ± 0.4	0.8 ± 0.3	0.6 ± 0.2	0.6 ± 0.2	*0.001*	0.7 ± 0.3
1-year after HTx	0.7 ± 0.4	0.9 ± 0.5	0.7 ± 0.3	0.8 ± 0.6	*0.390*	0.7 ± 0.4
3-year after HTx	0.7 ± 0.4	0.8 ± 0.3	0.7 ± 0.3	0.6 ± 0.2	*0.128*	0.7 ± 0.3

CPR*: cardiopulmonary resuscitation, cardiac massage during waiting before transplantation, E: ECMO, ECMO: extracorporeal membrane oxygenation, H/D: hemodialysis, HTx: heart transplantation, IABP: intra-aortic balloon pumping, ICMP: ischemic cardiomyopathy, MCS: mechanical circulatory support, SD: standard deviation, V: VAD, ventricular assist device
